# Clinical outcomes of interstitial lung abnormalities: a systematic review and meta-analysis

**DOI:** 10.1038/s41598-024-57831-3

**Published:** 2024-03-27

**Authors:** Jinwoo Seok, Shinhee Park, Eun Chong Yoon, Hee-Young Yoon

**Affiliations:** 1 Division of Allergy and Respiratory Diseases, Department of Internal Medicine, Soonchunhyang University Seoul Hospital, Seoul, 04401 Republic of Korea; 2 Division of Allergy and Respiratory Medicine, Department of Internal Medicine, Soonchunhyang University Bucheon Hospital, Bucheon, 14584 Republic of Korea

**Keywords:** Immune checkpoint inhibitors, Interstitial lung diseases, Lung neoplasms, Mortality, Radiation pneumonitis, Epidemiology, Risk factors, Respiratory signs and symptoms, Respiratory tract diseases

## Abstract

Interstitial lung abnormalities (ILA), incidental findings on computed tomography scans, have raised concerns due to their association with worse clinical outcomes. Our meta-analysis, which included studies up to April 2023 from PubMed/MEDLINE, Embase, and Cochrane Library, aimed to clarify the impact of ILA on mortality, lung cancer development, and complications from lung cancer treatments. Risk ratios (RR) with 95% confidence intervals (CI) were calculated for outcomes. Analyzing 10 studies on ILA prognosis and 9 on cancer treatment complications, we found that ILA significantly increases the risk of overall mortality (RR 2.62, 95% CI 1.94–3.54; I^2^ = 90%) and lung cancer development (RR 3.85, 95% CI 2.64–5.62; I^2^ = 22%). Additionally, cancer patients with ILA had higher risks of grade 2 radiation pneumonitis (RR 2.28, 95% CI 1.71–3.03; I^2^ = 0%) and immune checkpoint inhibitor-related interstitial lung disease (RR 3.05, 95% CI 1.37–6.77; I^2^ = 83%) compared with those without ILA. In conclusion, ILA significantly associates with increased mortality, lung cancer risk, and cancer treatment-related complications, highlighting the necessity for vigilant patient management and monitoring.

## Introduction

Interstitial lung disease (ILD) is characterized by inflammation and fibrosis of the lung interstitium, resulting in progressive lung damage and compromised respiratory function^1^. Within the spectrum of ILD, interstitial lung abnormalities (ILA) have emerged as incidental radiographic findings on computed tomography (CT) scans of the lungs^2^. The prevalence of ILA is 2–7% in the general population^3–7^ and 4–9% in individuals with a history of smoking^3,7–11^. Notably, a recent meta-analysis reported an ILA prevalence of 26% in familial pulmonary fibrosis cohorts^7^. Several risk factors have been proposed for the development of ILA, including advanced age, male sex, lower forced vital capacity (FVC)% predicted, smoking history, genetic mutations (e.g., *MUC5B*), and exposure to occupational and environmental pollutants^3,4,7,12–15^.

Several studies have reported an association between ILA and various clinical outcomes, including mortality, development of lung cancer, changes in lung function, and disease progression^3,16–19^. Additionally, there is increasing awareness regarding the influence of ILA on cancer treatment-related complications, including radiation therapy, immune checkpoint inhibitors (ICI), and surgery^20–22^. In 2020, the Fleischner Society standardized the definition of ILA as incidental findings on CT imaging, characterized by nondependent interstitial abnormalities involving more than 5% of any lung zone^2^. The standardized definition of ILA demonstrates the growing recognition of their distinct nature and emphasizes the importance of evaluating the clinical impact and implications of ILA for patient outcomes. Therefore, we aimed to conduct a comprehensive meta-analysis to thoroughly examine the association between ILA and various clinical outcomes such as mortality, development of lung cancer, and cancer treatment-related complications. We hypothesized that patients with ILA would demonstrate a poorer prognosis than those without ILA.

## Methods

### Literature search and study inclusion

This meta-analysis followed the guidelines outlined in the Preferred Reporting Items for Systematic Reviews and Meta-Analyses statement^23^. The study protocol was registered in PROSPERO (CRD42023437679). A thorough literature search was performed using electronic databases including PubMed/MEDLINE, Embase, and the Cochrane Library to identify relevant articles. The search encompassed articles published between the inception of these databases and April 2023. The search strategy was developed using appropriate keywords, and specific search strategies are provided in Supplementary Tables S1–S3. Furthermore, we included all relevant studies cited in a previous comprehensive review^2,24^. To ensure comprehensive coverage, we manually searched the reference lists of relevant original and review articles to identify additional eligible studies.

The inclusion criteria for the studies were as follows: (1) randomized controlled trials, cohort studies, or case–control studies evaluating ILA; (2) clinical outcomes including mortality, lung cancer development, changes in lung function, and lung cancer treatment-related complications; (3) studies written in English; and (4) participants aged ≥ 18 years. The exclusion criteria were as follows: (1) animal studies or in vitro studies; (2) case reports or case series with a small sample size (less than 20); (3) conference abstracts or posters without full-text availability; and (4) duplicate studies.

While we conducted the literature search together, it should be noted that the studies included to examine cancer treatment-related complications focused exclusively on patients with cancer. To avoid potential bias in evaluating the overall prognosis of ILA based solely on this subgroup, we separately analysed these studies to specifically assess the complications related to cancer treatment in patients with ILA. In our study, when multiple studies were included in a single paper and their results were reported separately, each study was treated as an individual entity for the analysis. Even if the studies or populations were the same, we considered them separate studies if there were changes in the study pool or if different outcomes were reported.

### Definition of ILA

According to the definition established by the Fleischner Society, ILA are defined as incidental findings on CT imaging, characterized by non-dependent changes that involve more than 5% of any lung zone, including ground-glass or reticular abnormalities, traction bronchiectasis, architectural distortion, honeycombing, and non-emphysematous cysts^2^. However, it is important to note that previous studies conducted before 2020 had different criteria for defining ILA, including additional lesions. Here, we provide detailed descriptions of the specific ILA definitions used in each study. In our study, “indeterminate ILA” referred to cases where radiological findings are consistent with ILA, but the extent of the lesion measures less than 5%^2,18^, although there is currently no universally accepted definition for it.

### Study design and quality assessment

Two independent reviewers screened the titles and abstracts of the identified articles based on the predetermined criteria. Full-text articles that met the eligibility criteria were assessed for inclusion. Disagreements between the reviewers were resolved through discussion, and a third reviewer was consulted if needed for consensus.

Data extraction from the included studies followed a standardized approach using a predefined form. The extracted information included the study characteristics, patient demographics, ILA characteristics, clinical outcomes (mortality, lung cancer development, hospitalization, and changes in lung function), and cancer treatment-related complications. To assess the cause of death, we categorized the outcomes into respiratory, cardiovascular (CV), and lung cancer-related mortalities. Cancer treatment-related outcomes, such as radiation pneumonitis (RP), immune checkpoint inhibitor-related interstitial lung disease (ICI-ILD), and postoperative pulmonary complications (PPC), were assessed using the Common Terminology Criteria for Adverse Events guidelines. RP severity was further classified into ≥ grade (Gr) 2 and ≥ Gr 3 for detailed analysis.

The quality of the included studies was assessed using the Newcastle–Ottawa Scale^25^, which evaluates selection (representativeness, selection of the non-exposed cohort, ascertainment of exposure, outcome of interest not present at start of study), comparability, and outcome (assessment of outcome, length of follow-up, and adequacy of follow-up). Scores higher than 7 indicate low risk of bias, scores ranging from 5 to 7 indicate moderate risk, and scores below 5 indicate high risk. Two independent reviewers conducted the assessment with a third-party arbitrator involved in resolving disagreements and ensuring consensus.

### Statistical analysis

To compare the prognosis between the two groups, the risk ratio (RR) with the corresponding 95% confidence interval (CI) was calculated for dichotomous outcomes. The heterogeneity of the included studies was assessed using the I^2^ statistic. I^2^ values ≤ 40% were considered insignificant, values from 30 to 60% indicated moderate heterogeneity, values from 50 to 90% denoted substantial heterogeneity, and values ≥ 75% indicated considerable heterogeneity. Due to the heterogeneity of the studies, a random-effects model was employed to estimate effect sizes. Subgroup analyses were performed based on the study population, specifically comparing the general population and high-risk group for lung cancer in terms of overall mortality. Subgroup analyses of the other outcomes could not be conducted because of the limited number of included studies. Sensitivity analyses for overall mortality were performed based on the study design (cohort vs. case–control) and ILA definition of the Fleischner Society. Publication bias was not evaluated using funnel plots, given the limited number of included studies (n < 10). Statistical significance was defined as a P value < 0.05. All data analyses were conducted using Review Manager version 5.4.1.

## Results

### Description of included studies

**Figure 1:**
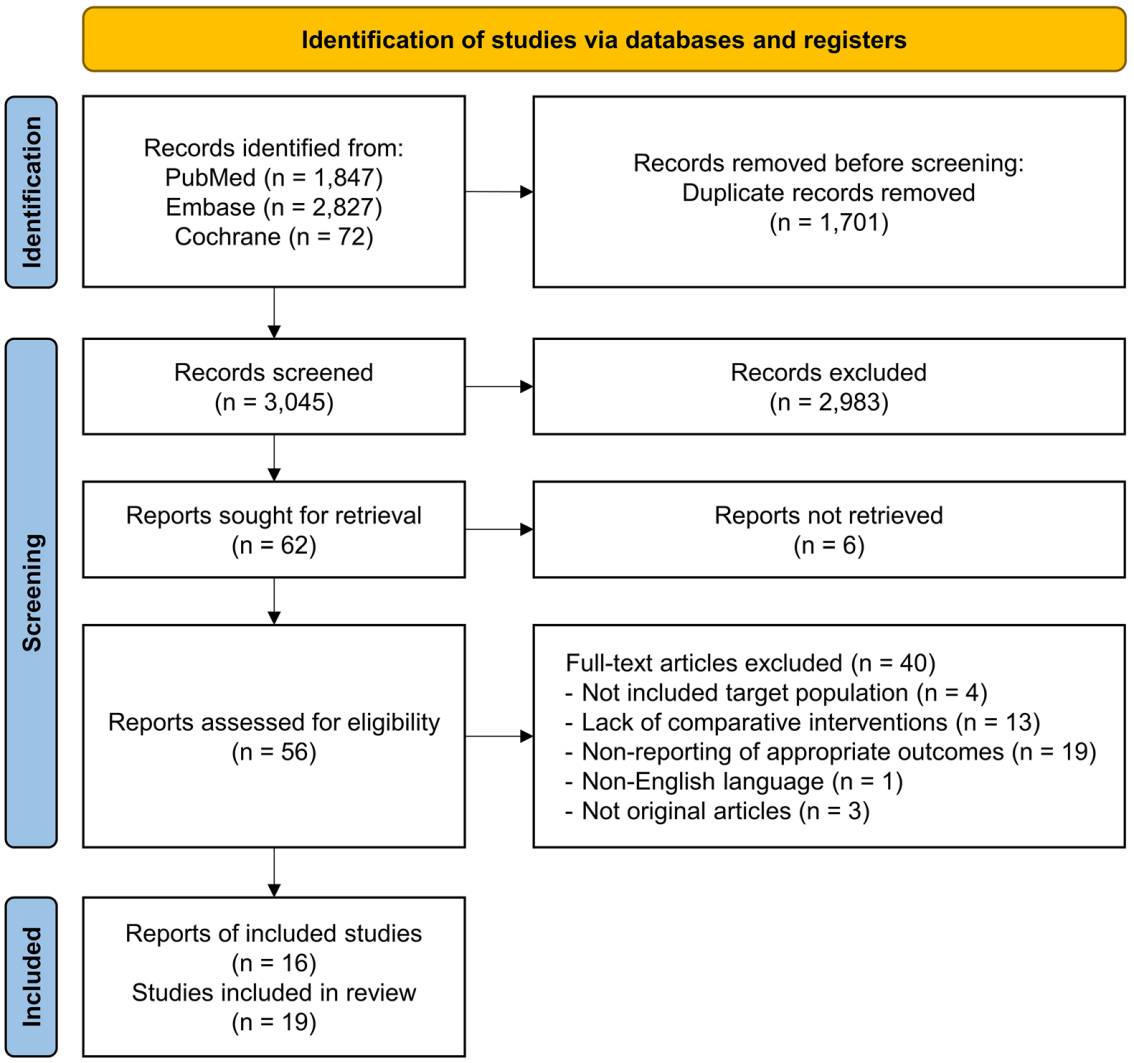
Preferred Reporting Items for Systematic Reviews and Meta-Analyses (PRISMA) flow diagram illustrating the study selection process.

The search identified 4746 records. After removing duplicates and screening, a total of 19 studies from 16 articles were included (Fig. 1). Of the 19 studies, 10 were studies on the prognosis of ILA, and the remaining 9 studies were on cancer treatment-related complications (Table 1).

**Table 1:** Characteristics of the included studies for prognosis and cancer treatment-related complications.

Study, year		Design	Site	Study population	ILA definition	Sample size^a^	Age, years	Male	Follow-up, year	Outcome
Prognosis										
Putman, 2016^3^	FHS	Cohort study	USA	Health screening	Compatible with Fleischner Society definition^b^, but including the presence of centrilobular nodularity lesion	2633 (177/1086)	ILA: 70 ± 12 Non-ILA: 56 ± 11	ILA: 88 (50) Non-ILA: 695 (51)	4 (3–5)	Mortality
	AGES-Reykjavik	Cohort study	Iceland	Birth cohort		5320 (378/1726)	ILA: 78 ± 6 Non-ILA: 76 ± 5	ILA: 206 (54) Non-ILA: 1306 (41)	9 (7–10)	Mortality
	COPDGene	Case–control	USA	Smoking		2068 (156/739)	ILA: 64 ± 9 Non-ILA: 60 ± 9	ILA: 76 (49) Non-ILA: 609 (52)	7 (7–7)	Mortality
	ECLIPSE	Cohort study	12 countries	Smoking		1670 (157/985)	ILA: 64 ± 8 Non-ILA: 62 ± 7	ILA: 116 (74) Non-ILA: 346 (66)	3 (3–3)	Mortality
Ash, 2017^9^	COPDGene	Case–control	USA	Smoking	Non-dependent changes affecting more than 10% of any lung zone, including reticular or GGA, diffuse centrilobular nodularity, non-emphysematous cysts, HC, or traction BE	8266 (1069/NR)	60 ± 9	4256 (52)	6 ± 2	Mortality
Hoyer, 2018^11^	DLCST	Cohort study	Denmark	Lung cancer high risk	GGA, HC, reticulation, pleural nodules, centrilobular nodules, paraseptal/subpleural nodules, mosaic attenuation, and mass	1990 (332/NR)	63 ± 6	956 (56)	12 (11–12)	Mortality, cause specific mortality, lung cancer incidence, hospitalization
Axelsson, 2020^26^	AGES-Reykjavik	Cohort study	Iceland	Birth cohort	Compatible with Fleischner Society definition^b^, but including the presence of centrilobular nodularity lesion	5270 (375/1712)	ILA: 78 ± 6 Non-ILA: 76 ± 5	ILA: 205 (55) Non-ILA: 1296 (41)	9 (7–10)	Lung cancer incidence
Lee 1, 2022^17^		Cohort study	South Korea	Heath screening	Compatible with Fleischner Society definition^b^	840 (55/NR)	59 ± 7	564 (67)	11 ± 1	Mortality, cause specific mortality, lung cancer incidence
Lee 2, 2022^18^		Cohort study	South Korea	Heath screening	Compatible with Fleischner Society definition^b^	2765 (94/119)	59 ± 7	2068 (75)	12 (11–13)	Mortality, cause specific mortality, lung cancer incidence
Patel, 2023^16^	CTLS	Case–control	USA	Smoking	Compatible with Fleischner Society definition^b^	1669 (41/101)	63 ± 6	956 (56)	6 ± 2	Mortality, cause specific mortality, lung cancer incidence, hospitalization
Cancer treatment-related complications										
Yamaguchi, 2014^27^		Retrospective cohort	Japan	Thoracic cancer with RTx	Compatible with Fleischner Society definition^b^, but including the presence of centrilobular nodularity lesion	62 (11)	69 (43–86)	57 (92)	12	RP ≥ Gr 2
Li, 2018^28^		Retrospective cohort	China	SCLC with RTx	Reticular abnormalities, traction BE, bilateral independent GGA, HC, and non-emphysematous cysts	95 (15)	61 (42–80)	85 (89)	13 (3–29)	RP ≥ Gr 2
Nakanishi, 2019^29^		Retrospective cohort	Japan	Advanced NSCLC with ICI	Compatible with Fleischner Society definition^b^, but including the presence of centrilobular nodularity lesion	83 (3)	68 (34–85)	133 (67)	0.3 (0–1)	ICI-ILD
Shimoji, 2020^30^		Retrospective cohort	Japan	Nonlung cancer with ICI	Compatible with Fleischner Society definition^b^, but including the presence of centrilobular nodularity lesion and without any limitations on their extent	199 (37)	66 (20–93)	58 (70)	NR	ICI-ILD
Daido, 2022^31^		Retrospective cohort	Japan	Locally advanced NSCLC with ICI after CRT	Compatible with Fleischner Society definition^b^, but including the presence of centrilobular nodularity lesion	148 (56)	74 (43–86)	106 (72)	NR	ICI-ILD
Im, 2022^22^^c^		Case–control	South Korea	Underwent curative lung resection	Compatible with Fleischner Society definition^b^	300 (50)	69 ± 7	266 (89)	4 (2–5)	PPC
Murata, 2022^20^		Retrospective cohort	Japan	Advanced or recurrent NSCLC with ICIs	Compatible with Fleischner Society definition^b^	264 (57)	70 (63–75)	109 (74)	1	ICI-ILD
Jeong, 2023^32^^d^		Retrospective cohort	South Korea	Unresectable NSCLC with RTx	Compatible with Fleischner Society definition^b^	201 (44)	65 ± 7	188 (94)	2 ± 1	RP ≥ Gr 2
Ito, 2023^33^^e^		Retrospective cohort	Japan	NSCLC with RTx	Compatible with Fleischner Society definition^b^	175 (64)	ILA: 72 (60–86) Non-ILA: 71 (41–60)	ILA: 52 (81) Non-ILA: 82 (78)	2 (1–10)	RP ≥ Gr 2

Data are expressed as mean ± standard deviation, median (interquartile range), or number (%).

^a^Number (ILA/indeterminate ILA) in studies on prognosis and number (ILA) in studies on cancer treatment-related complications. ^b^Incidental CT findings of non-dependent abnormalities, such as ground-glass or reticular abnormalities, lung distortion, traction bronchiectasis, honeycombing, and non-emphysematous cysts observed in more than 5% of any lung zone (i.e., upper, middle, and lower lung zones demarcated by the levels of the inferior aortic arch and right inferior pulmonary vein) during complete or partial chest CT examinations (e.g., abdominal or cardiac CT) where interstitial disease was not suspected. ^c^Included idiopathic pulmonary fibrosis (n = 50). ^d^Included indeterminate ILA (n = 24). ^e^Included ILD (n = 6).

*BE* bronchiectasis, *CRT* chemoradiotherapy, *CT* computed tomography, *GGA* ground-glass abnormalities, *Gr* grade, *HC* honeycombing, *ICI* immune checkpoint inhibitor, *ICI-ILD* immune checkpoint inhibitor-related interstitial lung disease, *ILA* interstitial lung abnormalities, *NR* not recorded, *NSCLC* non-small cell lung cancer, *PPC* postoperative pulmonary complications, *RP* radiation pneumonitis, *RTx* radiation therapy, *SCLC* small cell lung cancer.

The methodological quality of studies assessing the prognosis of ILA was high, with a mean score of 7.1 ± 0.7 (range 6–8), and 7 out of 10 studies scoring ≥ 7. However, studies on cancer treatment-related complications had a moderate overall methodological quality, with a mean score of 5.3 ± 1.4 (Supplementary Table S4).

Among ten studies on the prognosis of ILA^3,9,11,16–18,26^, five focused on the general population^3,17,18,26^, while the remaining five specifically studied high-risk groups for lung cancer, such as individuals who smoke heavily^3,9,11,16^. The sample sizes ranged from 840 to 5320, with follow-up durations ranging from 3 to 12 years. The prevalence of ILA varied across the populations studied. In the general population, the prevalence of ILA was reported to be 3–7%, while that of populations at a higher risk of developing lung cancer was 2% and 17%, respectively. Six studies included indeterminate ILA, with a prevalence ranging from 4 to 59%. The definitions of ILA used in these studies varied depending on whether they were published before or after the 2020 Fleischner Society definition. Some earlier studies included centrilobular nodules or used a disease extent of 10% in their definitions. We could not find comparative studies on pulmonary function in participants with and without ILA, and combining hospitalization data was not feasible because of methodological variations.

In nine studies on cancer treatment-related complications^20,22,27–33^, the prevalence of ILA ranged from 4 to 38%, and the follow-up periods varied from 0 to 12 years. Four studies adhered to the Fleischner Society definition, whereas the others had minor differences in ILA criteria. Four studies examined RP^27,28,32,33^, and another four studied ICI-ILD^20,29–31^. Notably, a single study focused on PPC^22^, which precluded the possibility of a comprehensive meta-analysis on PPC. A meta-analysis of the relationship between indeterminate ILA and cancer treatment-related complications was limited owing to the small number of studies.

### Impact of ILA on mortality

**Figure 2:**
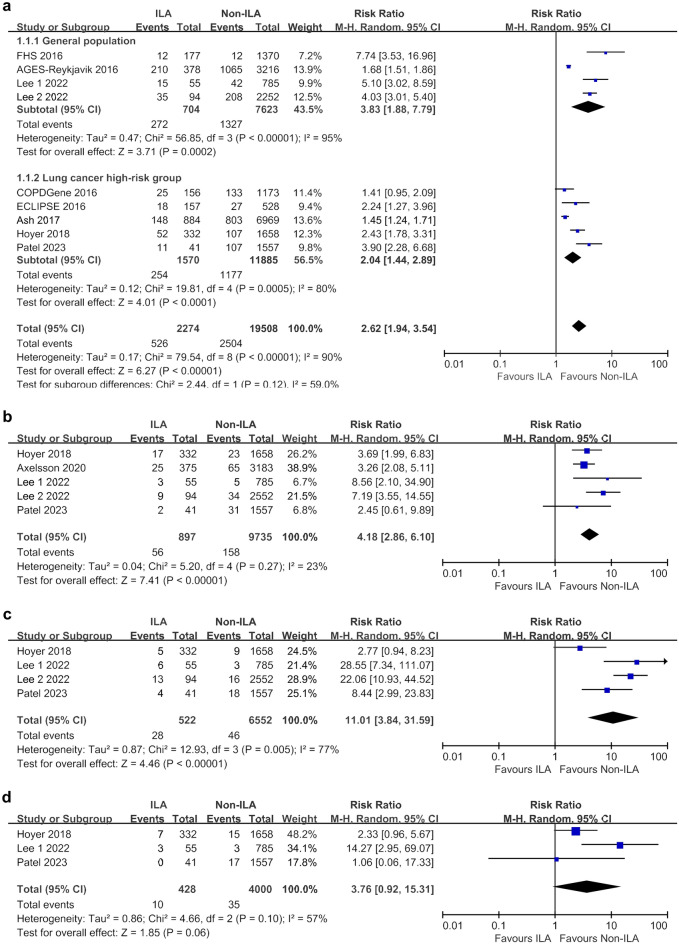
Meta-analyses of overall and cause-specific mortality between ILA and non-ILA groups. (**a**) Overall mortality; (**b**) Lung cancer-related mortality; (**c**) Respiratory disease-related mortality; (**d**) Cardiovascular disease-related mortality. *CI* confidence interval, *ILA* interstitial lung abnormalities, *M–H* Mantel–Haenszel method.

The ILA group showed a higher risk of overall mortality than the non-ILA group (RR 2.62, 95% CI 1.94–3.54), with significant heterogeneity (I^2^ = 90%). Subgroup analysis based on lung cancer risk consistently demonstrated increased overall mortality in the ILA group. Specifically, in the general population subgroup, the RR was even higher (RR 3.83, 95% CI 1.88–7.79), with greater heterogeneity (I^2^ = 95%) than that in the ILA group in the lung cancer risk population (RR 2.04, 95% CI 1.44–2.89; I^2^ = 80%) (Fig. 2a).

Regarding cause-specific mortality, significantly higher rates were observed in the ILA group than those in the non-ILA group for lung cancer-related mortality (RR 4.18, 95% CI 2.86–6.10; I^2^ = 23%) and respiratory-related mortality (RR 11.01, 95% CI 3.84–31.59; I^2^ = 77%) (Fig. 2b,c). However, the difference in CV-related mortality between the ILA and non-ILA groups did not reach statistical significance (RR 3.76, 95% CI 0.92–15.31; I^2^ = 57%) (Fig. 2d).

### Impact of indeterminate ILA on mortality

**Figure 3:**
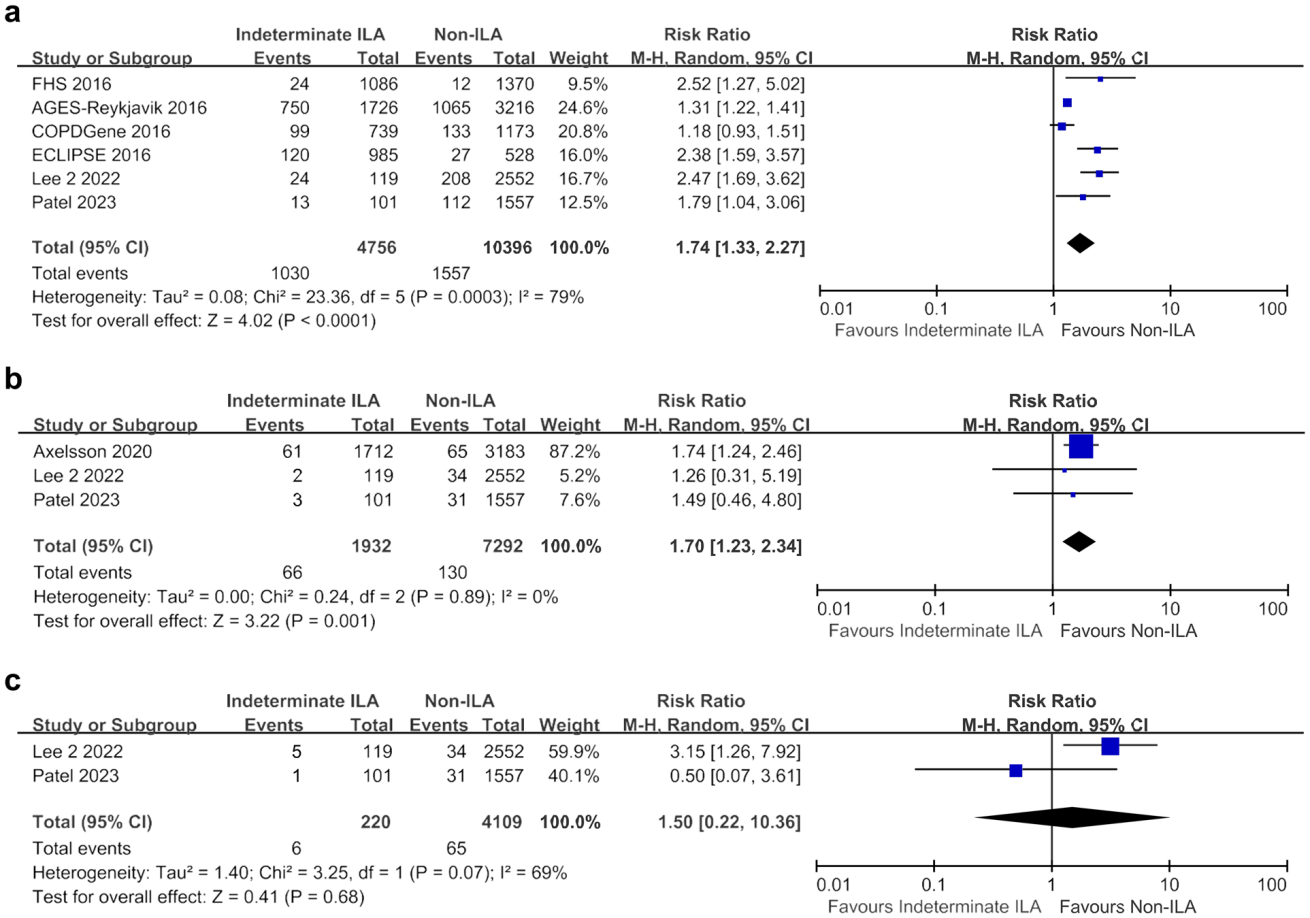
Meta-analyses of overall and cause-specific mortality between the indeterminate ILA and non-ILA groups. (**a**) Overall mortality; (**b**) Lung cancer-related mortality; (**c**) Respiratory disease-related mortality. *CI* confidence interval, *ILA* interstitial lung abnormalities, *M–H* Mantel–Haenszel method.

The indeterminate ILA group also exhibited higher mortality rates than the non-ILA group (RR 1.74, 95% CI 1.33–2.27; I^2^ = 79%) (Fig. 3a). Specifically, lung cancer-related mortality was significantly higher in the indeterminate ILA group than in the non-ILA group (RR 1.70, 95% CI 1.23–2.34; I^2^ = 0%) (Fig. 3b). However, there was no statistically significant difference in the risk of respiratory-related mortality between the two groups, although the RR was above 1 (RR 1.50, 95% CI 0.22–10.36; I^2^ = 69%) (Fig. 3c). CV-related mortality could not be included in the meta-analysis because of the limited data from only one study.

### Impact of ILA on lung cancer development

**Figure 4:**
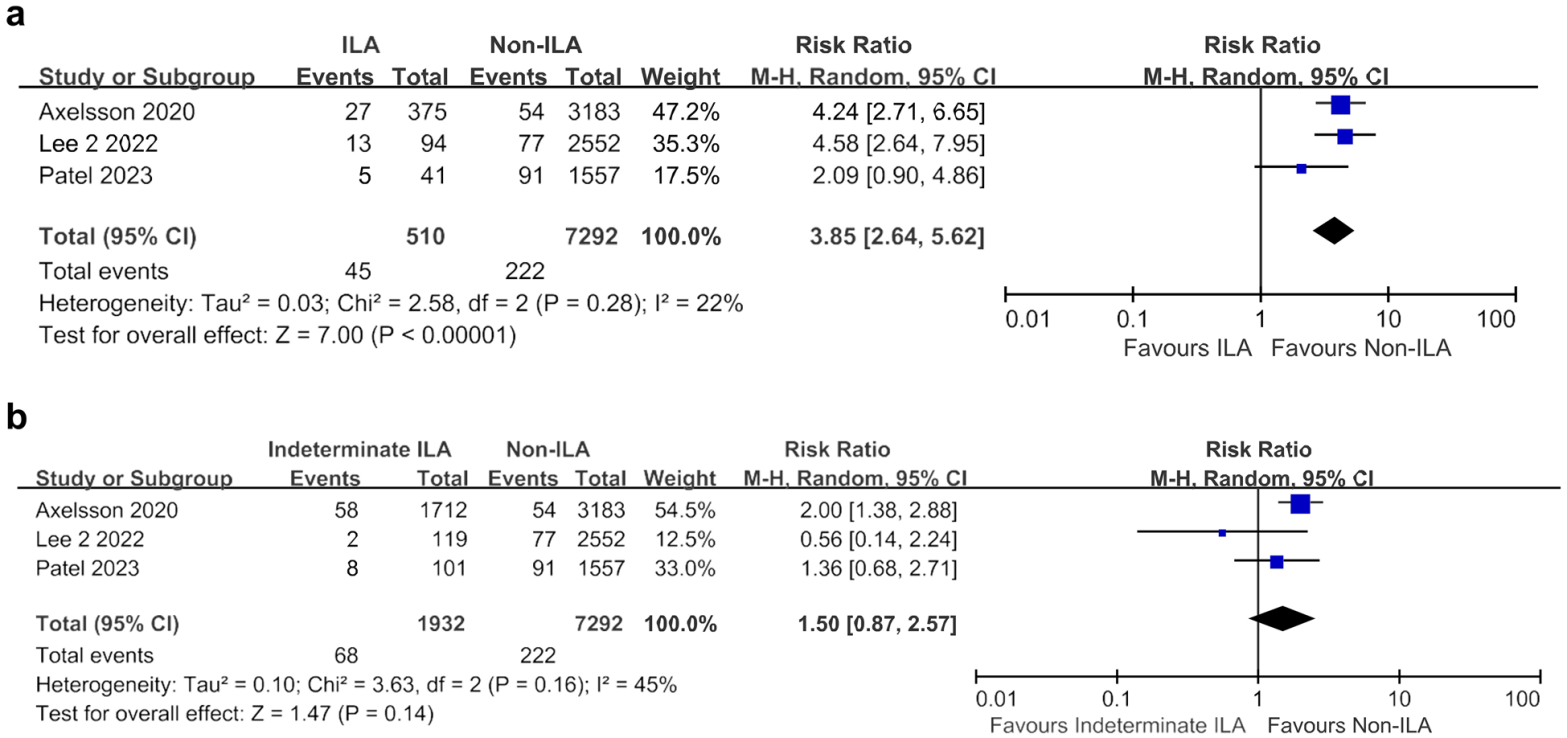
Meta-analyses of lung cancer development. (**a**) Between ILA and non-ILA groups; (**b**) Between indeterminate ILA and non-ILA groups. *CI* confidence interval, *ILA* interstitial lung abnormalities, *M–H* Mantel–Haenszel method.

ILA were associated with a significantly higher incidence of lung cancer compared to the non-ILA group (RR 3.85, 95% CI 2.64–5.62; I^2^ = 22%) based on the analysis of three studies (Fig. 4a). However, the indeterminate ILA group did not show a statistically significant increase in lung cancer risk compared to the non-ILA group (RR 1.50, 95% CI 0.87–2.57; I^2^ = 45%) (Fig. 4b).

### Impact of ILA on cancer treatment-related complications

**Figure 5:**
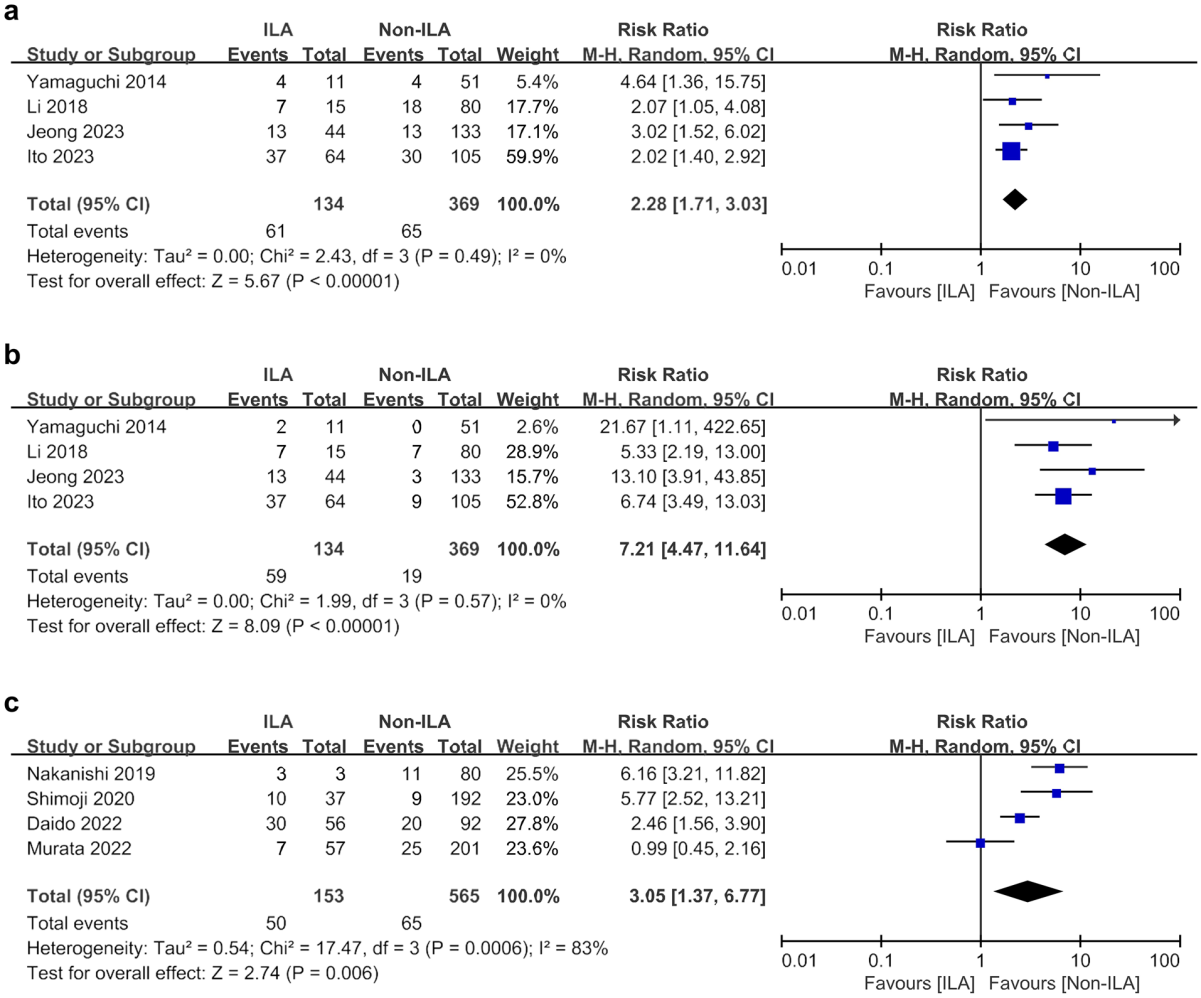
Meta-analyses of cancer treatment -related complications between ILA and non-ILA groups. (**a**) Radiation pneumonitis ≥ grade 2; (**b**) Radiation pneumonitis ≥ grade 3; (**c**) Immune checkpoint inhibitor-related interstitial lung disease. *CI* confidence interval, *ILA* interstitial lung abnormalities, *M–H* Mantel–Haenszel method.

In patients with lung cancer, the ILA group was found to be associated with a higher risk of ≥ Gr 2 RP (RR 2.28, 95% CI 1.71–3.03; I^2^ = 0%) and ≥ Gr 3 RP (RR 7.21, 95% CI 4.47–11.64; I^2^ = 0%) than the non-ILA group (Fig. 5a,b). ICI-ILD was significantly more common in the ILA group than in the non-ILA group (RR 3.05, 95% CI 1.37–6.77; I^2^ = 83%) (Fig. 5c).

### Sensitivity analyses for overall mortality

**Figure 6:**
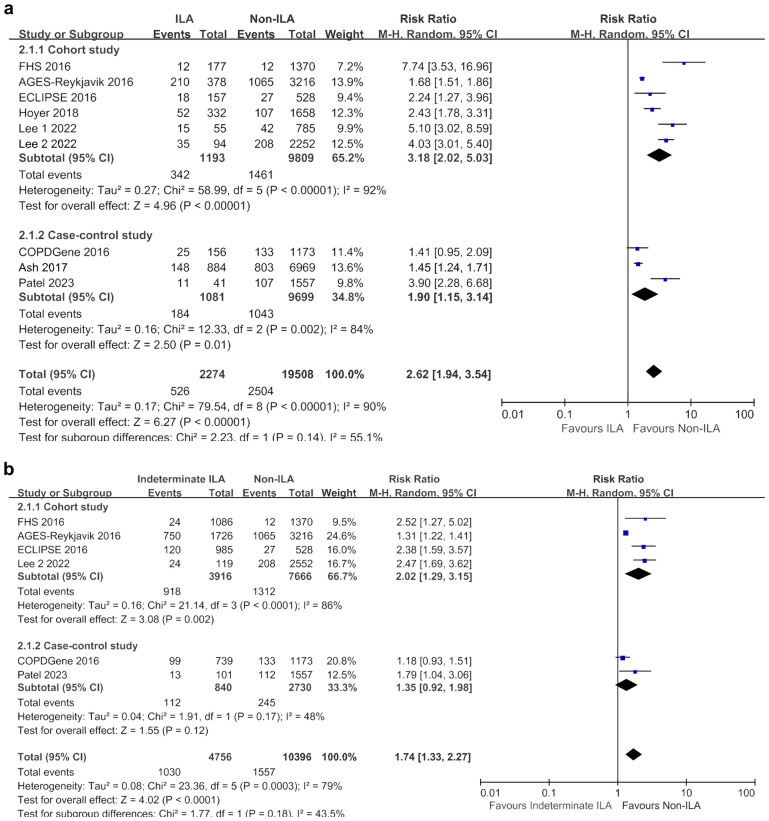
Sensitivity analyses based on study design for overall mortality. (**a**) Between ILA and non-ILA groups; (**b**) Between indeterminate ILA and non-ILA groups. *CI* confidence interval, *ILA* interstitial lung abnormalities, *M–H* Mantel–Haenszel method.

In the sensitivity analyses conducted by dividing according to study design, the observational studies (n = 6) indicated higher mortality in both ILA (RR 3.18, 95% CI 2.02–5.03; I^2^ = 92%) and indeterminate ILA (RR 2.02, 95% CI 1.29–3.15; I^2^ = 86%) groups compared with the non-ILA group (Fig. 6). However, in groups where only case–control studies were conducted (n = 4), ILA showed higher overall mortality compared with the non-ILA group (RR 1.90, 95% CI 1.15–3.14; I^2^ = 84%), while indeterminate ILA demonstrated only a tendency towards higher mortality (RR 1.35, 95% CI 0.92–1.98; I^2^ = 48%).

**Figure 7:**
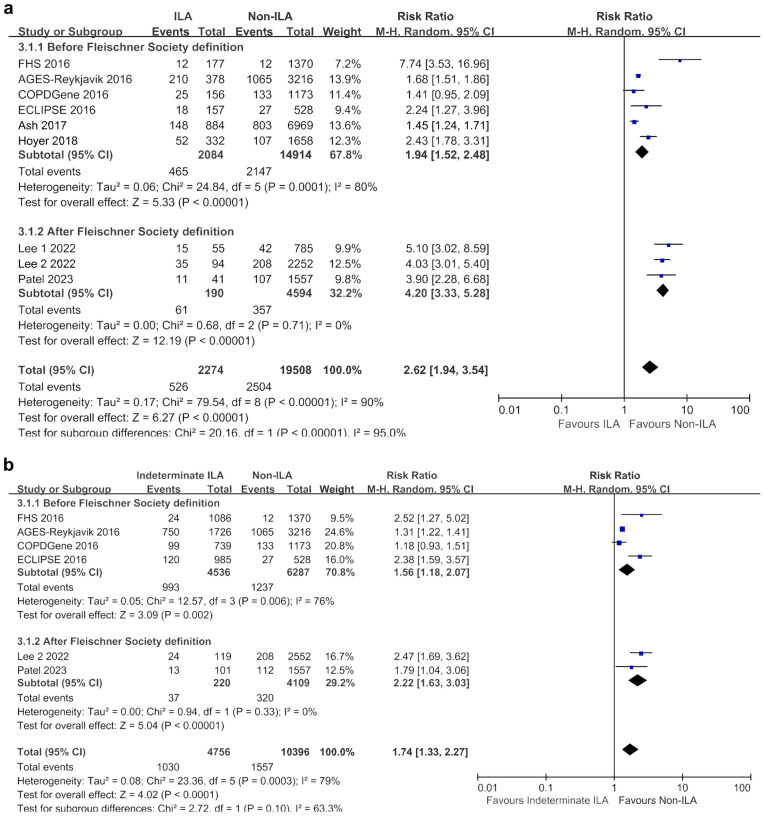
Sensitivity analyses of studies based on Fleischner Society definition of ILA for overall mortality. (**a**) Between ILA and non-ILA groups; (**b**) Between indeterminate ILA and non-ILA groups. *CI* confidence interval, *ILA* interstitial lung abnormalities, *M–H* Mantel–Haenszel method.

Regardless of adherence to the Fleischner Society's 2020 ILA definition, the ILA group exhibited higher mortality rates compared with the non-ILA group (2020 ILA definition: RR 4.20, 95% CI 3.33–5.28; I^2^ = 32.2%; non-2020 ILA definition: RR 1.94, 95% CI 1.52–2.48; I^2^ = 80.0%) (Fig. 7). Similarly, the indeterminate ILA group also showed higher mortality rates than the non-ILA group (2020 ILA definition: RR 2.22, 95% CI 1.63–3.03; I^2^ = 0%; non-2020 ILA definition: RR 1.56, 95% CI 1.18–2.07; I^2^ = 32.2%).

## Discussion

Our comprehensive meta-analysis, the first of its kind, demonstrates that ILA are associated with an elevated risk of overall mortality, with a notable increase in lung cancer and respiratory-related deaths, and an elevated incidence of lung cancer. Consistent results for overall mortality were observed in analyses based on study design and the 2020 ILA definition Fleischner Society. Our study also demonstrates that ILA are associated with a higher risk of RP and ICI-ILD in patients with lung cancer, and that indeterminate ILA are associated with higher overall and lung cancer mortality rates. This meta-analysis provides a systematic examination of ILA prognosis across various outcomes, making a valuable contribution to the existing literature.

Our findings are consistent with previous research demonstrating that ILA and indeterminate ILA are associated with increased mortality compared to non-ILA individuals^3,7,9,11,16–18^. Recent meta-analyses reported a higher pooled mortality risk in individuals with ILAs compared to those without (odds ratio (OR), 3.56; 95% CI 2.19–5.81)^7^, which aligns with our findings. However, our study extends its analysis by examining cause-specific mortality in the ILA, specifically identifying associations with lung cancer and respiratory-related causes of death. Although ILA are typically defined in the absence of respiratory symptoms or abnormal lung function, studies have shown associations between ILA and impaired exercise capacity and decline in pulmonary function^34–37^. Lee et al. found that among patients with chronic obstructive lung disease (n = 363), those with ILA were older, had lower forced expiratory volume in 1 s (FEV_1_) and FVC, and a significantly higher rate of annual decline in FEV_1_ and FVC in patients with progressive ILA than in those with stable or improved ILA^34^. In the AGES-Reykjavik cohort study (n = 375), Axelsson et al. reported that the presence of ILA was associated with decreased physical function, including decreased grip strength (OR 1.21, 95% CI 1.02–1.42), knee extension strength (OR 1.23, 95% CI 1.07–1.41), gait speed (OR 1.06, 95% CI 1.01–1.12), and thigh muscle mass (OR 1.14, 95% CI 1.05–1.23) in multivariable models^35^. These findings suggest that although ILA may not be identical to ILD, they can serve as early indicators of lung disease, thereby increasing mortality risk. Furthermore, the progression rate of ILA, which ranges from 6 to 80.5%, supports these results^5,6,8,38^. Park et al. reported that among Korean individuals with ILA who underwent consecutive chest CT scans for health screening (n = 200), 80.5% showed ILA progression with the median time to ILA progression being 3.2 years^38^. Our findings also align with the concept of ILA as early surrogate markers for respiratory diseases associated with higher mortality, especially in respiratory- and lung cancer-related deaths. Furthermore, differences in baseline characteristics such as age and comorbidities between the ILA and non-ILA groups may have influenced the higher mortality rates observed in the ILA group^3,39^. However, even after adjusting for these differences in several previous studies^3,9,11,16,39^, the ILA group consistently demonstrated a higher mortality rate, indicating that ILA themselves contribute to increased mortality. Furthermore, in our research, the heightened mortality observed in ILA cases may be linked to the progression of ILA to ILD. In an observational, retrospective multicenter study, 17% of fibrotic ILA cases (n = 59) and 6% of non-fibrotic ILA cases (n = 35) progressed to ILD over a median follow-up period of 12 years, with no instances of ILD progression during the same period observed in the non-ILA group (n = 2552) or the equivocal ILA group (n = 119)^18^. In another retrospective study of lung cancer patients who underwent surgical resection, six individuals developed ILD during follow-up: one with equivocal ILAs (4.5%) and five with fibrotic ILAs (19.2%), with no cases in the non-ILA group (n = 291) over a median follow-up period of 1313 days^40^. These findings collectively suggest in our study that the higher mortality rate observed in ILA cases, particularly the increased respiratory-related mortality, could be indicative of the potential for progression to ILD. Additionally, these findings suggest that ILA could serve as an early marker for ILD, emphasizing the clinical significance and stressing the importance of early detection and management in patients with these abnormalities.

In our study, ILA were found to affect the development of lung cancer. Smoking and age are well-established risk factors for lung cancer. The higher prevalence of individuals who smoke and older adults in the ILA group, which is consistent with previous studies^3,9,18,39^, could contribute to an increased risk of lung cancer. ILA and lung cancer may share other common risk factors, such as environmental exposure or air pollution^41,42^. Sack et al. reported an increased risk of ILA associated with self-reported vapour/gas exposure in currently employed individuals (OR 1.97, 95% CI 1.16–3.35) and those under 65 years old (OR 1.76, 95% CI 1.09–2.84) in the Multi-Ethnic Study of Atherosclerosis (MESA) cohort (n = 2312) during 10-year follow-up^14^. In addition, higher levels of ambient nitrogen oxide tended to increase the risk of ILA (OR 1.62, 95% CI 0.97–2.71; P = 0.06), particularly in individuals who do not smoke (OR 2.60, 95% CI 1.20–5.61; P = 0.02) in the MESA cohort (n = 6813)^43^. Additionally, the Framingham Heart Study showed an association between ILA and air pollution, specifically elemental carbon^15^. These shared risk factors may contribute to a higher incidence of lung cancer in patients with ILA.

We also found that ILA were significantly associated with the occurrence of RP and ICI-ILD in patients with cancer. Pre-existing ILD has been identified as a risk factor for RP or ICI-ILD development in previous studies^44–48^. Ueki et al. reported that the presence of ILD was significantly associated with grade ≥ 2 RP (hazard ratio 5.52, 95% CI 2.43–12.5) on multivariable analysis in patients with stage I non-small cell lung cancer (NSCLC) who underwent stereotactic body radiation therapy (n = 157)^47^. Yamaguchi et al. similarly demonstrated that pre-existing ILD was a significant risk factor for ICI-ILD on the multivariable model (OR 5.92, 95% CI 2.07–18.54) in 313 patients with cancer, including NSCLC (n = 96)^48^. Therefore, ILA, as surrogate markers of early ILD, may indicate an increased risk of treatment-related complications such as RP and ICI-ILD.

Our study has several limitations that should be acknowledged. First, the included studies exhibited heterogeneity in terms of study design, sample size, follow-up duration, and ILA definition, which may have introduced potential bias and affected the generalizability of the results. Specifically, our study, predominantly composed of cohort studies, was susceptible to biases due to this heterogeneity. To mitigate this issue, sensitivity analyses were conducted focusing on (observational vs. case–control studies), which showed consistent trends towards an increased risk of mortality in both ILA and indeterminate ILA patients. Although consistent trends were also found in sensitivity analyses focusing on the Fleischner Society definition, further meta-analyses that include recent studies following the standardized definition of ILA in 2020 would provide valuable insights. Second, the majority of the included studies focused on specific populations at a high risk of lung cancer, which may restrict the applicability of our findings to the general population. However, similar results were observed in the subgroup analyses focusing on the general population. Third, our analysis primarily focused on mortality outcomes, with limited data available on other important clinical parameters, such as pulmonary function decline, hospitalization, or development of ILD. Studies examining diverse outcomes will enhance our understanding of the implications of ILA. Lastly, our analysis predominantly used case–control and retrospective studies from East Asian countries, limiting generalization to other regions. Future research should include more diverse populations and prospective study designs to validate and expand our findings. Despite these limitations, our study provided valuable insights into the prognosis of patients with ILA.

## Conclusion

In conclusion, our study emphasizes the strong association between ILA and important clinical outcomes, such as mortality, lung cancer development, and cancer treatment-related complications. These findings underscore the importance of close monitoring and managing patients with ILA, as they may require personalized interventions and comprehensive care. Further research is required to better understand the underlying mechanisms and develop effective strategies for the prevention and treatment of ILA and their associated complications.

### Supplementary Information

Supplementary Tables.

## Data Availability

The datasets generated during and/or analysed during the current study are available from the corresponding author on reasonable request.
