# Supplementary material for: Clinical outcomes of interstitial lung abnormalities: a systematic review and meta-analysis
Source: Sci Rep. 2024 Mar 27;14:7330. doi: 10.1038/s41598-024-57831-3 (PMC10973382; doi:10.1038/s41598-024-57831-3)
Supplement: Supplementary file 1 — Supplementary Tables. [file 41598_2024_57831_MOESM1_ESM.docx]

**Clinical outcomes of interstitial lung abnormalities: a systematic review and meta-analysis**

**Jinwoo Seok^1†^, Shinhee Park^2†^, Eun Chong Yoon^1^, Hee-Young Yoon^1*^**

^1^Division of Allergy and Respiratory Diseases, Department of Internal Medicine, Soonchunhyang University Seoul Hospital, Seoul 04401, Republic of Korea.

^2^Division of Allergy and Respiratory Medicine, Department of Internal Medicine, Soonchunhyang University Bucheon Hospital, Bucheon 14584, Republic of Korea.

^*^email: yhyoung85@schmc.ac.kr

^†^Jinwoo Seok and Shinhee Park contributed equally to this work.

**Table S1.** Search strategy for PubMed/MEDLINE

| **Search number** | **Query** | **Search details** | **Results** |
| --- | --- | --- | --- |
| 1 | "abnormal CT finding*"[Title/Abstract] | "abnormal ct finding*"[Title/Abstract] | 251 |
| 2 | "early interstitial lung*"[Title/Abstract] | "early interstitial lung*"[Title/Abstract] | 23 |
| 3 | "early lung abnormal*"[Title/Abstract] | "early lung abnormal*"[Title/Abstract] | 4 |
| 4 | "evidence for ILD"[Title/Abstract] - Schema: all | "evidence for ILD"[Title/Abstract] | 0 |
| 5 | "idiopathic interstitial pneumonitis"[Title/Abstract] | "idiopathic interstitial pneumonitis"[Title/Abstract] | 74 |
| 6 | "interstitial abnormal*"[Title/Abstract] | "interstitial abnormal*"[Title/Abstract] | 144 |
| 7 | "interstitial lung abnormal*"[Title/Abstract] | "interstitial lung abnormal*"[Title/Abstract] | 247 |
| 8 | "lung attenuation*"[Title/Abstract] | "lung attenuation*"[Title/Abstract] | 241 |
| 9 | "pleural abnormal*"[Title/Abstract] | "pleural abnormal*"[Title/Abstract] | 282 |
| 10 | "preclinical ILD*"[Title/Abstract] | "preclinical ild*"[Title/Abstract] | 6 |
| 11 | "preclinical lung*"[Title/Abstract] | "preclinical lung*"[Title/Abstract] | 58 |
| 12 | "pulmonary abnormalit*"[Title/Abstract] | "pulmonary abnormalit*"[Title/Abstract] | 1,067 |
| 13 | "sub clinical lung*"[Title/Abstract] | "sub clinical lung*"[Title/Abstract] | 5 |
| 14 | "subclinical ILD*"[Title/Abstract] | "subclinical ild*"[Title/Abstract] | 24 |
| 15 | "subclinical inflammatory lung*"[Title/Abstract] - Schema: all | "subclinical inflammatory lung*"[Title/Abstract] | 0 |
| 16 | "subclinical interstitial lung*"[Title/Abstract] | "subclinical interstitial lung*"[Title/Abstract] | 56 |
| 17 | "subclinical lung*"[Title/Abstract] | "subclinical lung*"[Title/Abstract] | 58 |
| 18 | "subclinical parenchymal lung*"[Title/Abstract] | "subclinical parenchymal lung*"[Title/Abstract] | 2 |
| 19 | "unclassified interstitial pneumonia"[Title/Abstract] | "unclassified interstitial pneumonia"[Title/Abstract] | 7 |
| 20 | #1 OR #2 OR #3 OR #4 OR #5 OR #6 OR #7 OR #8 OR #9 OR #10 OR #11 OR #12 OR #13 OR #14 OR #15 OR #16 OR #17 OR #18 OR #19 | "abnormal ct finding*"[Title/Abstract] OR "early interstitial lung*"[Title/Abstract] OR "early lung abnormal*"[Title/Abstract] OR "idiopathic interstitial pneumonitis"[Title/Abstract] OR "interstitial abnormal*"[Title/Abstract] OR "interstitial lung abnormal*"[Title/Abstract] OR "lung attenuation*"[Title/Abstract] OR "pleural abnormal*"[Title/Abstract] OR "preclinical ild*"[Title/Abstract] OR "preclinical lung*"[Title/Abstract] OR "pulmonary abnormalit*"[Title/Abstract] OR "sub clinical lung*"[Title/Abstract] OR "subclinical ild*"[Title/Abstract] OR "subclinical interstitial lung*"[Title/Abstract] OR "subclinical lung*"[Title/Abstract] OR "subclinical parenchymal lung*"[Title/Abstract] OR "unclassified interstitial pneumonia"[Title/Abstract] | 2,445 |
| 21 | Mortality[MeSH Terms] | "mortality"[MeSH Terms] | 422,474 |
| 22 | mortalit*[Title/Abstract] | "mortalit*"[Title/Abstract] | 974,025 |
| 23 | #21 OR #22 | "mortality"[MeSH Terms] OR "mortalit*"[Title/Abstract] | 1,231,530 |
| 24 | Lung Injury[MeSH Terms] | "lung injury"[MeSH Terms] | 47,347 |
| 25 | "lung injur*"[Title/Abstract] | "lung injur*"[Title/Abstract] | 35,631 |
| 26 | #24 OR #25 | "lung injury"[MeSH Terms] OR "lung injur*"[Title/Abstract] | 69,324 |
| 27 | "lung damage*"[Title/Abstract] | "lung damage*"[Title/Abstract] | 5,101 |
| 28 | Pulmonary Valve Insufficiency[MeSH Terms] | "pulmonary valve insufficiency"[MeSH Terms] | 2,222 |
| 29 | "pulmonary valve insufficienc*"[Title/Abstract] | "pulmonary valve insufficienc*"[Title/Abstract] | 154 |
| 30 | "pulmonary insufficienc*"[Title/Abstract] | "pulmonary insufficienc*"[Title/Abstract] | 1,512 |
| 31 | #29 OR #30 | "pulmonary valve insufficienc*"[Title/Abstract] OR "pulmonary insufficienc*"[Title/Abstract] | 1,646 |
| 32 | #28 OR #31 | "pulmonary valve insufficiency"[MeSH Terms] OR "pulmonary valve insufficienc*"[Title/Abstract] OR "pulmonary insufficienc*"[Title/Abstract] | 3,589 |
| 33 | "lung insufficienc*"[Title/Abstract] | "lung insufficienc*"[Title/Abstract] | 40 |
| 34 | Lung Neoplasms[MeSH Terms] | "lung neoplasms"[MeSH Terms] | 271,658 |
| 35 | "lung cancer*"[Title/Abstract] | "lung cancer*"[Title/Abstract] | 202,189 |
| 36 | "lung neoplasm*"[Title/Abstract] | "lung neoplasm*"[Title/Abstract] | 9,044 |
| 37 | "lung tumo*"[Title/Abstract] | "lung tumo*"[Title/Abstract] | 16,626 |
| 38 | #35 OR #36 OR #37 | "lung cancer*"[Title/Abstract] OR "lung neoplasm*"[Title/Abstract] OR "lung tumo*"[Title/Abstract] | 216,991 |
| 39 | #34 OR #38 | "lung neoplasms"[MeSH Terms] OR "lung cancer*"[Title/Abstract] OR "lung neoplasm*"[Title/Abstract] OR "lung tumo*"[Title/Abstract] | 339,055 |
| 40 | Pneumonia[MeSH Terms] | "pneumonia"[MeSH Terms] | 318,496 |
| 41 | "lung inflammation*"[Title/Abstract] | "lung inflammation*"[Title/Abstract] | 8,851 |
| 42 | pneumonia[Title/Abstract] | "pneumonia"[Title/Abstract] | 151,487 |
| 43 | #41 OR #42 | "lung inflammation*"[Title/Abstract] OR "pneumonia"[Title/Abstract] | 159,709 |
| 44 | #40 OR #43 | "pneumonia"[MeSH Terms] OR "lung inflammation*"[Title/Abstract] OR "pneumonia"[Title/Abstract] | 405,770 |
| 45 | Lung Diseases, Interstitial[MeSH Terms] | "lung diseases, interstitial"[MeSH Terms] | 84,186 |
| 46 | "interstitial lung disease*"[Title/Abstract] | "interstitial lung disease*"[Title/Abstract] | 15,644 |
| 47 | #45 OR #46 | "lung diseases, interstitial"[MeSH Terms] OR "interstitial lung disease*"[Title/Abstract] | 90,144 |
| 48 | Lung Diseases[MeSH Terms] | "lung diseases"[MeSH Terms] | 1,201,698 |
| 49 | "lung disease*"[Title/Abstract] | "lung disease*"[Title/Abstract] | 71,598 |
| 50 | "pulmonary disease*"[Title/Abstract] | "pulmonary disease*"[Title/Abstract] | 82,255 |
| 51 | #49 OR #50 | "lung disease*"[Title/Abstract] OR "pulmonary disease*"[Title/Abstract] | 144,531 |
| 52 | #48 OR #51 | "lung diseases"[MeSH Terms] OR "lung disease*"[Title/Abstract] OR "pulmonary disease*"[Title/Abstract] | 1,247,591 |
| 53 | "lung fibros*"[Title/Abstract] | "lung fibros*"[Title/Abstract] | 5,535 |
| 54 | "lung function*"[Title/Abstract] | "lung function*"[Title/Abstract] | 41,609 |
| 55 | "pulmonary function*"[Title/Abstract] | "pulmonary function*"[Title/Abstract] | 37,366 |
| 56 | "lung mechanic*"[Title/Abstract] | "lung mechanic*"[Title/Abstract] | 2,654 |
| 57 | "cancer of the lung*"[Title/Abstract] | "cancer of the lung*"[Title/Abstract] | 1,895 |
| 58 | "upper lobe fibros*"[Title/Abstract] | "upper lobe fibros*"[Title/Abstract] | 47 |
| 59 | cancerization*[Title/Abstract] | "cancerization*"[Title/Abstract] | 1,418 |
| 60 | outcome[Title/Abstract] | "outcome"[Title/Abstract] | 1,244,802 |
| 61 | #23 OR #26 OR #27 OR #32 OR #33 OR #39 OR #44 OR #47 OR #52 OR #53 OR #54 OR #55 OR #56 OR #57 OR #58 OR #59 OR #60 | "mortality"[MeSH Terms] OR "mortalit*"[Title/Abstract] OR "lung injury"[MeSH Terms] OR "lung injur*"[Title/Abstract] OR "lung damage*"[Title/Abstract] OR "pulmonary valve insufficiency"[MeSH Terms] OR "pulmonary valve insufficienc*"[Title/Abstract] OR "pulmonary insufficienc*"[Title/Abstract] OR "lung insufficienc*"[Title/Abstract] OR "lung neoplasms"[MeSH Terms] OR "lung cancer*"[Title/Abstract] OR "lung neoplasm*"[Title/Abstract] OR "lung tumo*"[Title/Abstract] OR "pneumonia"[MeSH Terms] OR "lung inflammation*"[Title/Abstract] OR "pneumonia"[Title/Abstract] OR "lung diseases, interstitial"[MeSH Terms] OR "interstitial lung disease*"[Title/Abstract] OR "lung diseases"[MeSH Terms] OR "lung disease*"[Title/Abstract] OR "pulmonary disease*"[Title/Abstract] OR "lung fibros*"[Title/Abstract] OR "lung function*"[Title/Abstract] OR "pulmonary function*"[Title/Abstract] OR "lung mechanic*"[Title/Abstract] OR "cancer of the lung*"[Title/Abstract] OR "upper lobe fibros*"[Title/Abstract] OR "cancerization*"[Title/Abstract] OR "outcome"[Title/Abstract] | 3,503,563 |
| 62 | #20 AND #61 | ("abnormal ct finding*"[Title/Abstract] OR "early interstitial lung*"[Title/Abstract] OR "early lung abnormal*"[Title/Abstract] OR "idiopathic interstitial pneumonitis"[Title/Abstract] OR "interstitial abnormal*"[Title/Abstract] OR "interstitial lung abnormal*"[Title/Abstract] OR "lung attenuation*"[Title/Abstract] OR "pleural abnormal*"[Title/Abstract] OR "preclinical ild*"[Title/Abstract] OR "preclinical lung*"[Title/Abstract] OR "pulmonary abnormalit*"[Title/Abstract] OR "sub clinical lung*"[Title/Abstract] OR "subclinical ild*"[Title/Abstract] OR "subclinical interstitial lung*"[Title/Abstract] OR "subclinical lung*"[Title/Abstract] OR "subclinical parenchymal lung*"[Title/Abstract] OR "unclassified interstitial pneumonia"[Title/Abstract]) AND ("mortality"[MeSH Terms] OR "mortalit*"[Title/Abstract] OR ("lung injury"[MeSH Terms] OR "lung injur*"[Title/Abstract]) OR "lung damage*"[Title/Abstract] OR ("pulmonary valve insufficiency"[MeSH Terms] OR ("pulmonary valve insufficienc*"[Title/Abstract] OR "pulmonary insufficienc*"[Title/Abstract])) OR "lung insufficienc*"[Title/Abstract] OR ("lung neoplasms"[MeSH Terms] OR ("lung cancer*"[Title/Abstract] OR "lung neoplasm*"[Title/Abstract] OR "lung tumo*"[Title/Abstract])) OR ("pneumonia"[MeSH Terms] OR ("lung inflammation*"[Title/Abstract] OR "pneumonia"[Title/Abstract])) OR ("lung diseases, interstitial"[MeSH Terms] OR "interstitial lung disease*"[Title/Abstract]) OR ("lung diseases"[MeSH Terms] OR ("lung disease*"[Title/Abstract] OR "pulmonary disease*"[Title/Abstract])) OR "lung fibros*"[Title/Abstract] OR "lung function*"[Title/Abstract] OR "pulmonary function*"[Title/Abstract] OR "lung mechanic*"[Title/Abstract] OR "cancer of the lung*"[Title/Abstract] OR "upper lobe fibros*"[Title/Abstract] OR "cancerization*"[Title/Abstract] OR "outcome"[Title/Abstract]) | 1,847 |

**Table S2.** Search strategy for Embase

| **Search number** | **Query** | **Search details** | **Results** |
| --- | --- | --- | --- |
| 1 | 'abnormal ct finding*':ab,ti | 'abnormal ct finding*':ab,ti | 380 |
| 2 | 'early interstitial lung*':ab,ti | 'early interstitial lung*':ab,ti | 40 |
| 3 | 'early lung abnormal*':ab,ti | 'early lung abnormal*':ab,ti | 7 |
| 4 | 'evidence for ild':ab,ti | 'evidence for ild':ab,ti | 6 |
| 5 | 'idiopathic interstitial pneumonitis':ab,ti | 'idiopathic interstitial pneumonitis':ab,ti | 110 |
| 6 | 'interstitial abnormal*':ab,ti | 'interstitial abnormal*':ab,ti | 206 |
| 7 | 'interstitial lung abnormal*':ab,ti | 'interstitial lung abnormal*':ab,ti | 383 |
| 8 | 'lung attenuation*':ab,ti | 'lung attenuation*':ab,ti | 381 |
| 9 | 'pleural abnormal*':ab,ti | 'pleural abnormal*':ab,ti | 386 |
| 10 | 'preclinical ild*':ab,ti | 'preclinical ild*':ab,ti | 10 |
| 11 | 'preclinical lung*':ab,ti | 'preclinical lung*':ab,ti | 104 |
| 12 | 'pulmonary abnormalit*':ab,ti | 'pulmonary abnormalit*':ab,ti | 1,320 |
| 13 | 'sub clinical lung*':ab,ti | 'sub clinical lung*':ab,ti | 12 |
| 14 | 'subclinical ild*':ab,ti | 'subclinical ild*':ab,ti | 70 |
| 15 | 'subclinical inflammatory lung*':ab,ti | 'subclinical inflammatory lung*':ab,ti | 1 |
| 16 | 'subclinical interstitial lung*':ab,ti | 'subclinical interstitial lung*':ab,ti | 92 |
| 17 | 'subclinical lung*':ab,ti | 'subclinical lung*':ab,ti | 87 |
| 18 | 'subclinical parenchymal lung*':ab,ti | 'subclinical parenchymal lung*':ab,ti | 3 |
| 19 | 'unclassified interstitial pneumonia':ab,ti | 'unclassified interstitial pneumonia':ab,ti | 8 |
| 20 | #1 OR #2 OR #3 OR #4 OR #5 OR #6 OR #7 OR #8 OR #9 OR #10 OR #11 OR #12 OR #13 OR #14 OR #15 OR #16 OR #17 OR #18 OR #19 | 'abnormal ct finding*':ab,ti OR 'early interstitial lung*':ab,ti OR 'early lung abnormal*':ab,ti OR 'evidence for ild':ab,ti OR 'idiopathic interstitial pneumonitis':ab,ti OR 'interstitial abnormal*':ab,ti OR 'interstitial lung abnormal*':ab,ti OR 'lung attenuation*':ab,ti OR 'pleural abnormal*':ab,ti OR 'preclinical ild*':ab,ti OR 'preclinical lung*':ab,ti OR 'pulmonary abnormalit*':ab,ti OR 'sub clinical lung*':ab,ti OR 'subclinical ild*':ab,ti OR 'subclinical inflammatory lung*':ab,ti OR 'subclinical interstitial lung*':ab,ti OR 'subclinical lung*':ab,ti OR 'subclinical parenchymal lung*':ab,ti OR 'unclassified interstitial pneumonia':ab,ti | 3,426 |
| 21 | 'mortality'/exp | 'mortality'/exp | 1,362,771 |
| 22 | mortalit*:ab,ti | mortalit*:ab,ti | 1,423,187 |
| 23 | #21 OR #22 | 'mortality'/exp OR mortalit*:ab,ti | 1,880,990 |
| 24 | 'lung injury'/exp | 'lung injury'/exp | 44,434 |
| 25 | 'lung injur*':ab,ti | 'lung injur*':ab,ti | 48,798 |
| 26 | 'lung damage*':ab,ti | 'lung damage*':ab,ti | 7,313 |
| 27 | #25 OR #26 | 'lung injur*':ab,ti OR 'lung damage*':ab,ti | 54,420 |
| 28 | #24 OR #27 | 'lung injury'/exp OR ('lung injur*':ab,ti OR 'lung damage*':ab,ti) | 72,870 |
| 29 | 'pulmonary valve insufficiency'/exp | 'pulmonary valve insufficiency'/exp | 4,822 |
| 30 | 'pulmonary valve insufficienc*':ab,ti | 'pulmonary valve insufficienc*':ab,ti | 172 |
| 31 | #29 OR #30 | 'pulmonary valve insufficiency'/exp OR 'pulmonary valve insufficienc*':ab,ti | 4,875 |
| 32 | 'lung insufficiency'/exp | 'lung insufficiency'/exp | 5,417 |
| 33 | 'pulmonary insufficienc*':ab,ti | 'pulmonary insufficienc*':ab,ti | 2,079 |
| 34 | 'lung insufficienc*':ab,ti | 'lung insufficienc*':ab,ti | 55 |
| 35 | #33 OR #34 | 'pulmonary insufficienc*':ab,ti OR 'lung insufficienc*':ab,ti | 2,133 |
| 36 | #32 OR #35 | 'lung insufficiency'/exp OR ('pulmonary insufficienc*':ab,ti OR 'lung insufficienc*':ab,ti) | 6,690 |
| 37 | 'lung cancer'/exp | 'lung cancer'/exp | 471,599 |
| 38 | 'lung cancer*':ab,ti | 'lung cancer*':ab,ti | 301,784 |
| 39 | #37 OR #38 | 'lung cancer'/exp OR 'lung cancer*':ab,ti | 516,604 |
| 40 | 'lung tumor'/exp | 'lung tumor'/exp | 540,496 |
| 41 | 'lung neoplasm*':ab,ti | 'lung neoplasm*':ab,ti | 1,524 |
| 42 | 'lung tumo*':ab,ti | 'lung tumo*':ab,ti | 24,256 |
| 43 | #41 OR #42 | 'lung neoplasm*':ab,ti OR 'lung tumo*':ab,ti | 25,567 |
| 44 | #40 OR #43 | 'lung tumor'/exp OR ('lung neoplasm*':ab,ti OR 'lung tumo*':ab,ti) | 543,178 |
| 45 | 'pneumonia'/exp | 'pneumonia'/exp | 399,342 |
| 46 | 'lung inflammation*':ab,ti | 'lung inflammation*':ab,ti | 12,714 |
| 47 | pneumonia:ab,ti | pneumonia:ab,ti | 219,664 |
| 48 | #46 OR #47 | 'lung inflammation*':ab,ti OR pneumonia:ab,ti | 231,498 |
| 49 | #45 OR #48 | 'pneumonia'/exp OR ('lung inflammation*':ab,ti OR pneumonia:ab,ti) | 452,511 |
| 50 | 'interstitial lung disease'/exp | 'interstitial lung disease'/exp | 121,479 |
| 51 | 'interstitial lung disease*':ab,ti | 'interstitial lung disease*':ab,ti | 27,256 |
| 52 | #50 OR #51 | 'interstitial lung disease'/exp OR 'interstitial lung disease*':ab,ti | 124,394 |
| 53 | 'lung disease'/exp | 'lung disease'/exp | 2,076,857 |
| 54 | 'lung disease*':ab,ti | 'lung disease*':ab,ti | 104,892 |
| 55 | 'pulmonary disease*':ab,ti | 'pulmonary disease*':ab,ti | 117,763 |
| 56 | #54 OR #55 | 'lung disease*':ab,ti OR 'pulmonary disease*':ab,ti | 209,814 |
| 57 | #53 OR #56 | 'lung disease'/exp OR ('lung disease*':ab,ti OR 'pulmonary disease*':ab,ti) | 2,100,891 |
| 58 | 'lung fibrosis'/exp | 'lung fibrosis'/exp | 96,241 |
| 59 | 'lung fibros*':ab,ti | 'lung fibros*':ab,ti | 9,019 |
| 60 | #58 OR #59 | 'lung fibrosis'/exp OR 'lung fibros*':ab,ti | 97,342 |
| 61 | 'lung function'/exp | 'lung function'/exp | 160,517 |
| 62 | 'lung function*':ab,ti | 'lung function*':ab,ti | 70,062 |
| 63 | 'pulmonary function*':ab,ti | 'pulmonary function*':ab,ti | 58,566 |
| 64 | #62 OR #63 | 'lung function*':ab,ti OR 'pulmonary function*':ab,ti | 118,669 |
| 65 | #61 OR #64 | 'lung function'/exp OR ('lung function*':ab,ti OR 'pulmonary function*':ab,ti) | 207,697 |
| 66 | 'lung mechanics'/exp | 'lung mechanics'/exp | 5,363 |
| 67 | 'lung mechanic*':ab,ti | 'lung mechanic*':ab,ti | 3,799 |
| 68 | #66 OR #67 | 'lung mechanics'/exp OR 'lung mechanic*':ab,ti | 6,880 |
| 69 | 'cancer of the lung*':ab,ti | 'cancer of the lung*':ab,ti | 2,449 |
| 70 | 'upper lobe fibros*':ab,ti | 'upper lobe fibros*':ab,ti | 80 |
| 71 | cancerization*:ab,ti | cancerization*:ab,ti | 1,746 |
| 72 | outcome:ab,ti | outcome:ab,ti | 1,794,209 |
| 73 | #23 OR #28 OR #31 OR #36 OR #39 OR #44 OR #49 OR #52 OR #57 OR #60 OR #65 OR #68 OR #69 OR #70 OR #71 OR #72 | ('mortality'/exp OR mortalit*:ab,ti) OR ('lung injury'/exp OR ('lung injur*':ab,ti OR 'lung damage*':ab,ti)) OR ('pulmonary valve insufficiency'/exp OR 'pulmonary valve insufficienc*':ab,ti) OR ('lung insufficiency'/exp OR ('pulmonary insufficienc*':ab,ti OR 'lung insufficienc*':ab,ti)) OR ('lung cancer'/exp OR 'lung cancer*':ab,ti) OR ('lung tumor'/exp OR ('lung neoplasm*':ab,ti OR 'lung tumo*':ab,ti)) OR ('pneumonia'/exp OR ('lung inflammation*':ab,ti OR pneumonia:ab,ti)) OR ('interstitial lung disease'/exp OR 'interstitial lung disease*':ab,ti) OR ('lung disease'/exp OR ('lung disease*':ab,ti OR 'pulmonary disease*':ab,ti)) OR ('lung fibrosis'/exp OR 'lung fibros*':ab,ti) OR ('lung function'/exp OR ('lung function*':ab,ti OR 'pulmonary function*':ab,ti)) OR ('lung mechanics'/exp OR 'lung mechanic*':ab,ti) OR 'cancer of the lung*':ab,ti OR 'upper lobe fibros*':ab,ti OR cancerization*:ab,ti OR outcome:ab,ti | 5,238,875 |

**Table S3.** Search strategy for the Cochrane Library

| **Search number** | **Query** | **Results** |
| --- | --- | --- |
| 1 | "abnormal CT finding*":ab,ti | 3 |
| 2 | "early interstitial lung*":ab,ti | 4 |
| 3 | "early lung abnormal*":ab,ti | 0 |
| 4 | "evidence for ILD":ab,ti | 0 |
| 5 | "idiopathic interstitial pneumonitis":ab,ti | 3 |
| 6 | "interstitial abnormal*":ab,ti | 4 |
| 7 | "interstitial lung abnormal*":ab,ti | 13 |
| 8 | "lung attenuation*":ab,ti | 12 |
| 9 | "pleural abnormal*":ab,ti | 6 |
| 10 | "preclinical ILD*":ab,ti | 0 |
| 11 | "preclinical lung*":ab,ti | 3 |
| 12 | "pulmonary abnormalit*":ab,ti | 30 |
| 13 | "sub clinical lung*":ab,ti | 1 |
| 14 | "subclinical ILD*":ab,ti | 4 |
| 15 | "subclinical inflammatory lung*":ab,ti | 0 |
| 16 | "subclinical interstitial lung*":ab,ti | 6 |
| 17 | "subclinical lung*":ab,ti | 7 |
| 18 | "subclinical parenchymal lung*":ab,ti | 0 |
| 19 | "unclassified interstitial pneumonia":ab,ti | 0 |
| 20 | #1 OR #2 OR #3 OR #4 OR #5 OR #6 OR #7 OR #8 OR #9 OR #10 OR #11 OR #12 OR #13 OR #14 OR #15 OR #16 OR #17 OR #18 OR #19 | 86 |
| 21 | Mesh Descriptor [Mortality], explode all tree | 21850 |
| 22 | mortalit*:ab,ti | 82335 |
| 23 | #21 OR #22 | 94337 |
| 24 | Mesh Descriptor [Lung Injury] explode all tree | 1994 |
| 25 | ("lung injur*"):ab,ti | 2213 |
| 26 | #24 OR #25 | 3561 |
| 27 | ("lung damage*):ab,ti | 288 |
| 28 | Mesh Descriptor [Pulmonary Valve Insufficiency] explode all tree | 169 |
| 29 | (pulmonary next valve insufficienc*):ab,ti | 5 |
| 30 | (pulmonary next insufficienc*):ab,ti | 64 |
| 31 | #29 OR #30 | 68 |
| 32 | #28 OR #31 | 237 |
| 33 | (lung next insufficienc*):ab,ti | 5 |
| 34 | Mesh Descriptor [Lung Neoplasms] explode all tree | 10306 |
| 35 | (lung next cancer*):ab,ti | 21039 |
| 36 | (lung next neoplasm*):ab,ti | 40 |
| 37 | (lung next tumo*):ab,ti | 329 |
| 38 | #35 OR #36 OR #37 | 21223 |
| 39 | #34 OR #38 | 23519 |
| 40 | Mesh Descriptor [Pneumonia] explode all tree | 9755 |
| 41 | (lung next inflammation*):ab,ti | 314 |
| 42 | pneumonia*):ab,ti | 14238 |
| 43 | #41 OR #42 | 14517 |
| 44 | #40 OR #43 | 19900 |
| 45 | Mesh Descriptor [Lung Diseases, Interstitial] explode all tree | 1528 |
| 46 | (interstitial lung disease*):ab,ti | 1298 |
| 47 | #45 OR #46 | 2488 |
| 48 | Mesh Descriptor [Lung Diseases] explode all tree | 57392 |
| 49 | (lung next disease*):ab,ti | 6123 |
| 50 | (pulmonary next disease*):ab,ti | 14458 |
| 51 | #49 OR #50 | 19510 |
| 52 | #48 OR #51 | 68892 |
| 53 | (lung next fibros*):ab,ti | 209 |
| 54 | (lung next function*):ab,ti | 11454 |
| 55 | (pulmonary next function*):ab,ti | 8883 |
| 56 | (lung next mechanic*):ab,ti | 339 |
| 57 | (cancer next of next the next lung*):ab,ti | 81 |
| 58 | (upper next lobe next fibros*):ab,ti | 0 |
| 59 | cancerization*):ab,ti | 102 |
| 60 | outcome: ab,ti | 450681 |
| 61 | #23 OR #26 OR #27 OR #32 OR #33 OR #39 OR #44 OR #47 OR #52 OR #53 OR #54 OR #55 OR #56 OR #57 OR #58 OR #59 OR #60 | 582353 |
| 62 | #20 AND #61 | 72 |

**Table S4.** Quality assessment of included studies using the nine-star Newcastle-Ottawa Scale

| **Study, year** | **Selection** | | | | **Comparability** | **Outcome** | | | **Total Stars** |
| --- | --- | --- | --- | --- | --- | --- | --- | --- | --- |
|  | **Representativeness of the exposed cohort** | **Selection of the non-exposed cohort** | **Ascertainment of exposure** | **Outcome of interest not present at start of study** | **Comparability of cohorts on the basis of the design or analysis** | **Assessment of outcome** | **Long enough follow-up** | **Adequacy of follow-up** |  |
| Prognosis | | | | | | | | | |
| FHS, 2016 | * | * | * | * | * | * | * | * | 8 |
| AGES-Reykjavik, 2016 | * | * | * | * | * | * | * | * | 8 |
| COPDGene, 2016 |  | * | * | * | * | * | * | * | 7 |
| ECLIPSE, 2016 |  | * | * | * | * | * | * |  | 6 |
| Ash, 2017 |  | * | * | * | * | * | * | * | 7 |
| Hoyer, 2018 |  | * | * | * | * | * | * | * | 7 |
| Axelsson, 2020 | * | * | * | * | * | * | * | * | 8 |
| Lee 1, 2022 | * | * | * | * | * | * | * |  | 7 |
| Lee 2, 2022 | * | * | * | * | * | * | * |  | 7 |
| Patel, 2023 |  | * | * | * | * | * | * |  | 6 |
| Cancer treatment-related complications | | | | | | | | | |
| Yamaguchi, 2014 |  | * | * | * |  | * | * |  | 5 |
| Li, 2018 |  | * |  | * | ** | * | * |  | 6 |
| Nakanishi, 2019 |  | * | * | * |  |  |  | * | 4 |
| Shimoji, 2020 |  | * | * | * |  |  |  | * | 4 |
| Daido, 2022 |  | * | * | * |  | * |  | * | 5 |
| Im, 2022 |  | * | * | * | ** | * | * | * | 8 |
| Murata, 2022 |  | * | * | * |  |  |  | * | 4 |
| Jeong, 2023 |  | * | * | * |  | * | * |  | 5 |
| Ito, 2023 |  | * | * | * | * | * | * | * | 7 |

A study can receive a maximum of one star for each numbered item within the selection and outcome categories, and a maximum of two stars for the comparability category.
